# Endosuture trainer box simulator as a tool for training and teaching in bariatric laparoscopic surgery

**DOI:** 10.1186/s12893-018-0412-5

**Published:** 2018-10-09

**Authors:** Luiz Gonzaga de Moura Júnior, Paulo Roberto Leitão de Vasconcelos, Francisco Vagnaldo Fechine, Mayra Sabiá de Moura, Régis Luiz Sabiá de Moura, Hermano Alexandre Lima Rocha, Manoel Odorico de Moraes Filho

**Affiliations:** 1 Centro Universitário Unichristus, R. João Adolfo Gurgel, 133 – Cocó, Fortaleza, CE CEP: 60190-060 Brazil; 2 Federal University of Ceara, Rua Prof. Costa Mendes, 1608 - Rodolfo Teófilo, Fortaleza, CE CEP 60430-140 Brazil; 3 Núcleo do Obeso do Ceará, Av. Antônio Sales, 1540 - Joaquim Távora, Fortaleza, CE 60135-101 Brazil; 4 Universidade de Fortaleza, Avenida Washington Soares, 1321 - Reitoria - Edson Queiroz, Fortaleza, CE 60811-905 Brazil; 5 Community Health Department. Rua Prof. Costa Mendes, Federal University of Ceará, 1608, 60, Fortaleza, Ceará 430-130 Brazil; 6 Federal Universityof Ceará – Rua Prof. Costa Mendes, 1608, 60, Fortaleza, Ceará 430-130 Brazil

**Keywords:** Laparoscopy, Surgery simulation, Resident education, Minimally invasive surgery

## Abstract

**Background:**

Video surgery requires acquisition of psychomotor skills that are different from those required for open surgery. The aim of this study was to assess the EndoSuture Trainer Box Simulator (ESTBS), a new bariatric laparoscopic skills simulator, as a tool for surgical education, comparing it with a standard laparoscopic trainer (SLT).

**Methods:**

A randomized prospective crossover study was designed to compare ESTBS versus SLT as a tool for training bariatric laparoscopic skills. Participants were assigned to perform a task simulating Nissen fundoplication operation. All subjects evaluated the simulators concerning to their performance on simulating laparoscopic procedures by the use of a questionnaire comparing: triangulation, resistance and resilience, spatial perception (stereotaxy), ergonomics and positioning, inverted movements, visibility, design, technical and technological resources for training and education. The overall score was defined as the median value obtained. A total of 37 participants were enrolled in the study, including 29 experienced surgeons (78.37%) and 08 surgical residents (21.63%).

**Results:**

A superior performance was observed with ESTBS as compared to SLT upon 7 of the 10 items evaluated in the questionnaire. Additionally, the overall score of ESTBS (median of 4, very good) was significantly higher (*P* < 0.0001) than that of SLT (median of 3, good). For the items, triangulation, resistance and resilience, ergonomics, design, training, technology and teaching, the evaluation for the ESTBS was very good and excellent, which was significantly higher than obtained by SLT. Also, ESTBS was cheaper.

**Conclusions:**

The ESTBS was shown to present excellent technical and technological performances and appears to constitute a useful cost-effective promising instrument for teaching and training bariatric surgical laparoscopic skills.

## Background

Using technical and instrumental training with simulators, the ability of surgical trainees has been assessed. It has shown improvement in teaching capabilities, minimizing the difficulties found at conventional clinical surgical curriculum teaching. Palter et al. [1] studied the effects the basic learning curve and the proficiency of virtual reality-based training, and watched the transfer of the learning curve in the simulation of laparoscopic procedures performed at a skills surgery laboratory (SSL) as compared to the operating room (OR). Residents trained with simulators showed superior technical ability and proficiency as compared to conventional surgical resident training. Video surgery requires acquisition of psychomotor skills that are different from those required for open surgery, such as: limited surgical field, fulcrum effect (inverted movements, taking as a fulcrum lever the abdominal wall), limited force response, absence of three-dimensional vision and modified spatial perception of depth (stereotaxy). High-fidelity models are employed for psychomotor and the ability of spatial vision abilities development on the outside (external interface) surgery educational training in order to acquire adequate proficiency to a satisfactory performance at real time in surgical patients.

Korndorffer et al. [2] catalogued and researched 253 general surgery programs in the United States and assessed the prevalence, utilization, equipments, types of training, supervision and cost of various operating skills laboratories. From 162 general surgery training programs surveyed, 64 utilized their complete training with SSL. The authors concluded that the vast majority of programs considered important and a requirement the use of SSL, whereas 45 of the programs did not have surgical simulator facilities.

Through the simulator it is possible to understand the basic procedures of laparoscopy, to validate the technical skills, to integrate and to compensate this lack of sufficient training in the surgical curriculum. Moura Jr. et al. [3] have developed a simulator for laparoscopic surgery upon the abdominal cavity – the EndoSuture Trainer Box Simulator (ESTBS), to apply it as an instrument of video training at a surgical teaching platform aiming to evolve and to promote technological innovation. The ESTBS was conceived with the support of architectural design, graphics design, electrical engineering and mechatronics. A patent application has been deposited at the Brazilian Institute of Industrial Property under the number 1301198280. This was possible due to the experience and clinical observation gained through the handling of several other models of simulators, demonstrations of ESTBS prototypes at surgical congresses and as well as a result of the implementation of extensive continuing education training laparoscopic courses. Progressive stations simulations utilized were based upon extensive training using a SSL model with the objective of stepping up its methodology as proposed in this study.

Thus, considering that the ESTBS can be a new useful device for training in the context of laparoscopic simulation surgical teaching, based on the concepts of video surgery (ergonomics, stereotaxy, ambidexterity, inverted movements and haptic touch), the purpose of this study was to validate the ESTB as well as to assess it as a tool for surgical education, comparing it with a standard utilized laparoscopic trainer (SLT).

## Methods

### EndoSuture trainer box simulator

The ESTBS consists of a fiberglass console, similar to human stem, a hollow ring suitable for surgical anteroposterior movements with the space created corresponding to a pneumoperitoneal ambient with access to an appropriate suture tray matrix to carry on procedures and sutures, at a similar depth to that found to reach abdominal structures by operative tactic maneuvers. It has got a docking device and an infrared high definition camera with zoom and light source is used. LED strips are employed and external management and stem anatomical handling is possible through the use of one of the portals. It can be coupled to LED TV 19 ‘-85 ‘or a set of video surgery with the use of two or three dimension cameras.

**Fig. 1:**
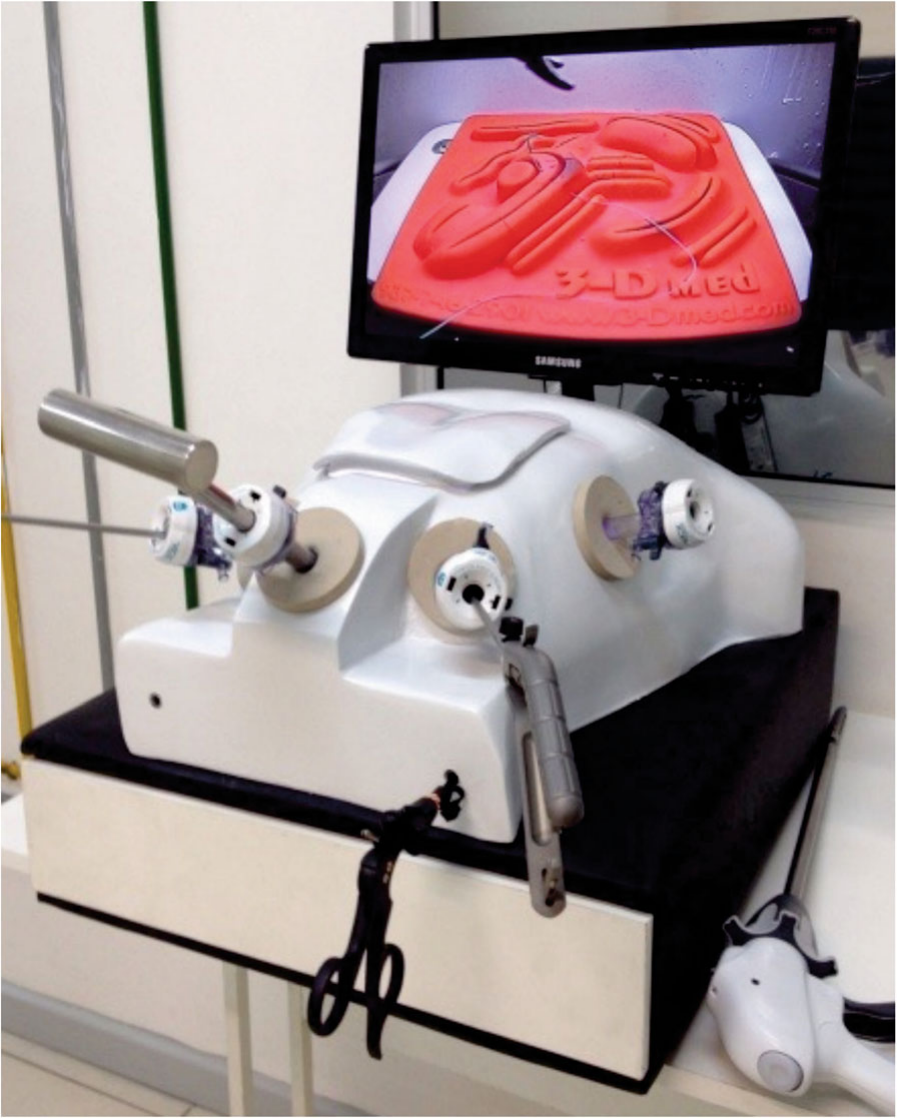
EndoSuture Trainer Box Simulator (ESTBS)

Laparoscopic portals are used to access the cavity through trocarters and instruments. It provides management by the surgeon with the aid of image movements, with proper distances that allow triangulation and convergence for adequate implementation of the endosuture. A laparoscopic, epigastric and a coupled window has a plug set in the “abdominal wall” of the simulator for full execution of the training under two-dimensional vision. Upon removable it allows direct vision (three dimensional) of the instruments on stage inverted movements exercises, resistance and resilience, ambidexterity and depth perception (stereotaxy). That apparatus has electrical and electronic components (LED lamp fixed in the inner abdominal like layer “peritoneum” of the mannequin, for lighting and satisfactory visibility of filming), plug electrical wiring, control cable, image stabilizer, energy current light switch, TV and camera) (Fig. 1).

Using simple materials and replacing specific devices such as monitors and camera to generic one allow an expressive reduction in cost compared to the standard laparoscopic trainer. The estimated cost of one full unit was one thousand dollars.

### Standard laparoscopic trainer

**Fig. 2:**
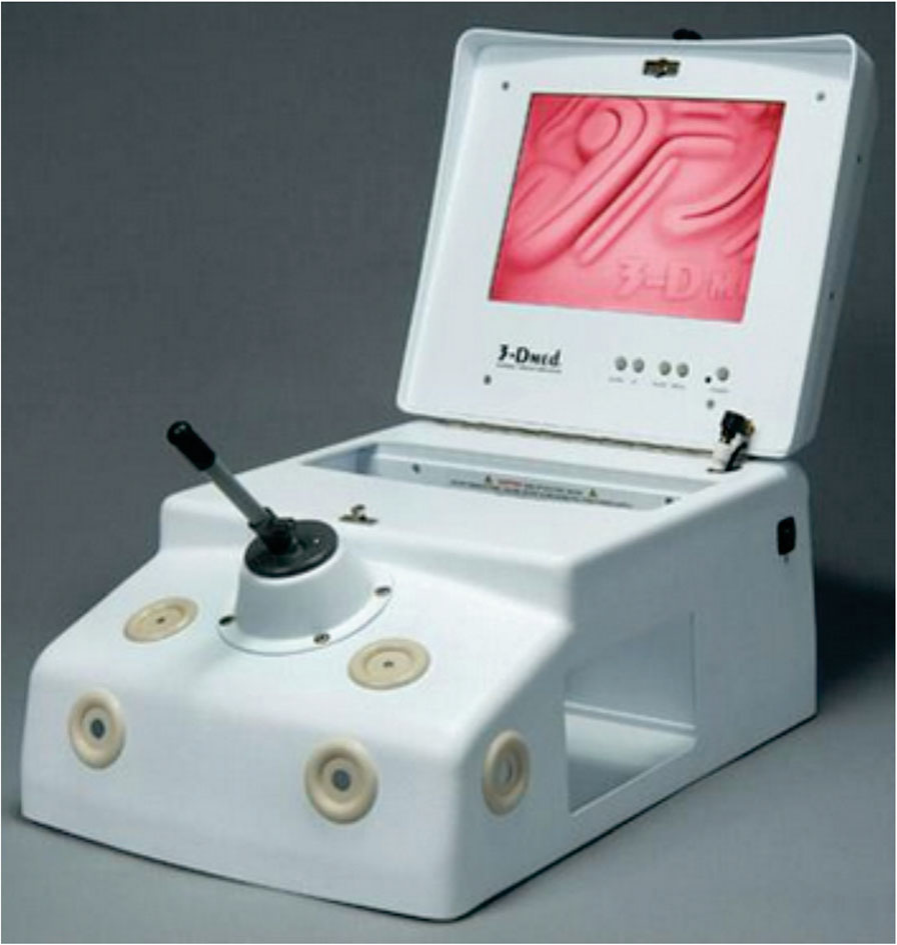
3-DMedⓇ T3 Joystick – Standard laparoscopic trainer (SLT)

The Standard Laparoscopic Trainer (SLT) used as the standard equipment for comparison in the present study has got a 3-DMed T3 Joystick^Ⓡ^ (3-DMed Medical Training Simulators, Franklin, Ohio, USA) and a Minimally Invasive Training System (MITS). It is simple to set up and portable and includes the original classic box, SimScope™ (camera) and a 10″ monitor. The device has 6 laparoscopic ports with interchangeable grommets (5 and 10 mm) and interior lighting. The instrument is designed to be used for one-person laparoscopic suturing workstation (Fig. 2). The cost of this equipment is $2850.00, according to the website of the Enterprise.

#### Study design and participants

The study carried using an SSL at Cesar Cals General Hospital, Fortaleza, Ceará, Brazil, and it has been approved by Ethics Committee of the Cesar Cals General Hospital / SES / SUS (#710.694). All participants provided a written informed consent after having received an explanation of the study design. They were recruited among members of Brazilian College of Surgeons and residents of general surgery and urology from the institution.

The study was conducted using a randomized prospective crossover study design to assess the ESTBS regarding its qualities as a tool for training laparoscopic skills, comparing it with the SLT. Therefore, a task was defined to assess laparoscopic skills, simulating the knot tying Nissen fundoplication operation (2 knots in diaphragmatic pillar and 4 knots in gastric fund, corresponding to 6 laparoscopic surgical knots).

Before performing the task on the simulators, the participants completed a questionnaire based on demographics and their previous laparoscopic experience, as well as received a general introduction to the ESTBS and SLT simulators. They were given a standardized and thorough explanation of the task, including a video demonstration.

The task consisted of performing a suture with 5 ties at each surgical knot in a soft tissue suture pad (3-DMed), using silk thread 2–0, or achieve a time limit of 18 min. Participants were assigned to perform the task using both the ESTBS and SLT consecutively. The starting order of simulators was randomized for each participant, using randomization software (Random Program line code – System Pause).

Before the testing period on surgical instruments at both simulators, a 10-min warm-up period was given to each participant. On completion of the task, all participants self-evaluated the technical and technological profile of the simulators and their ability of simulating laparoscopic procedures. After completing the task, they responded to a questionnaire containing 10 items based on a 5-point Likert scale, with scores from 1 to 5: 1. Insufficient; 2. Regular; 3. Good; 4. Very good; 5. Excellent. The following items were analyzed: 1. Triangulation of movements; 2. Resistance and resilience of portals; 3. Stereotaxy; 4. Ergonomics and positioning; 5. Fulcrum effect; 6. Visibility; 7. Design; 8. Technical resources for training; 9. Technological resource; 10. Teaching. The overall score was defined as the median of the 10 items.

Subsequently, surgeons were stratified on a progressive scale of skill and proficiency in suture video surgery, according to the time of task execution: 1. Insufficient (execution of 1, 2 or 3 knots in 18 min); 2. Regular (execution of 4 or 5 knots in 18 min); 3. Good (06 knots between 15 and 18 min); 4. Very good (06 knots between 12 and 15 min); 5. Excellent (06 knots execution in less than 12 min).

#### Statistical analysis

Descriptive statistics included the calculation of the mean and standard deviation for continuous variables, median and interquartile range for ordinal variables and absolute and relative frequencies for categorical variables. Comparisons between the two simulators regarding the scores of each item of the questionnaire were carried out using the Wilcoxon signed rank test. At all analyses, the significance level was set at 0.05 (5%). The software GraphPad Prism® version 5.00 (GraphPad Software, San Diego, California, USA, 2007) was used to perform the statistical procedures.

## Results

A total of 37 participants with different levels of laparoscopic expertise were recruited for this study, aged 39,7 years in average, with a standard deviation of 11,3, of whom, 13 (35.14%) were general surgeons, 8 (21.63%) general surgery and urology residents, and the others were experts surgeons in other surgical areas, who had at least 100 h of practice (43.23%). Demographics characteristics of the participants are shown in Table 1. Mean age was 39.70 years with predominance of males (91.89%) and right handed (94.59%).

**Table 1:** Characteristics of the participants of the study

Number of participants	37
Age (years): mean ± standard deviation	39.70 ± 11.33
Gender	
Male	34 (91.89%)
Female	3 (8.11%)
Dominant hand	
Right	35 (94.59%)
Left	2 (5.41%)
Specialty	
General Surgery	13 (35.14%)
Digestive tract surgery	3 (8.11%)
Bariatric Surgery	2 (5.41%)
Thoracic surgery	2 (5.41%)
Cardiac surgery	1 (2.70%)
Oncological surgery	1 (2.70%)
Pediatric Surgery	1 (2.70%)
Colorectal Surgery	2 (5.41%)
Gynecology	3 (8.11%)
Urology	1 (2.70%)
General surgery resident (R2)	6 (16.22%)
Urology resident (R1)	2 (5.41%)

The participants had assessed their performance of the two training devices in simulating the laparoscopic procedures (Nissen fundoplication operation knot tying) using a 10-item questionnaire based on a 5-point Likert scale (Table 2). It was found a superior performance of ESTBS as compared to SLT, concerning to: 1. triangulation of the instruments (*P* = 0.0001); 2. feedback of resistance (force applied on the instruments to move the leverage of the portals in the execution of tasks) and resiliency of the rubber silicone-grommets (spontaneous return of portals in original location after execution of movements) (*P* = 0.0005); 4. ergonomics of positioning (*P* = 0.0042); 7. simulators design (*P* < 0.0001); 8. technical resource to incorporate the training of the surgeons (P < 0.0001); 9. structural features to incorporate technology in training (P < 0.0001); 10. performance and effectiveness as a tool for training and retention of skills (teaching) (*P* = 0.0006). The two devices were similar in relation to: 3. perception of depth of intra-corporeal suture tray (stereotaxy) (*P* = 0.1627); 5. fulcrum effect (reverse motion) relative to real vision (*P* = 0.1500); and 6. visibility of the operating field and image quality monitor (*P* = 0.0650). The median overall score showed that the overall performance of ESTBS (4, very good) was significantly higher (P < 0.0001) than that of SLT (3, good).

**Table 2:** Validation of the EndoSuture Box Trainer Simulator (ESTBS) according to the analysis of the ratings given to both ESTBS and Standard Laparoscopic Trainer (SLT) by the 37 participants of the study for the 10 items of the questionnaire and the overall score, which was defined as the median of the 10. items. Comparisons between the scores of ESTBS and SLT were made using the Wilcoxon signed rank.test.

Items	Standard Laparoscopic Trainer (SLT)		EndoSuture Trainer Box Simulator (ESTBS)		Significance (Wilcoxon test)
	Median	Intequartile range	Median	Intequartile range	
1. Triangulation	3.00	2.50–4.00	4.00	3.00–4.00	P = 0.0001
2. Resistance	3.00	2.00–4.00	4.00	3.00–5.00	P = 0.0005
3. Stereotaxy	3.00	3.00–4.00	3.00	3.00–4.00	P = 0.1627
4. Ergonomics	3.00	2.00–3.50	4.00	3.00–4.00	P = 0.0042
5. Fulcrum	3.00	2.50–4.00	3.00	3.00–4.00	P = 0.1500
6. Visibility	3.00	3.00–4.00	4.00	3.00–4.00	P = 0.0650
7. Design	3.00	2.50–4.00	4.00	4.00–5.00	P < 0.0001
8. Training	3.00	2.00–3.50	4.00	3.00–4.00	*P* < 0.0001
9. Technology	3.00	2.00–3.50	4.00	3.00–4.50	P < 0.0001
10. Teaching	3.00	3.00–4.00	4.00	4.00–5.00	P = 0.0006
Overall score	3.00	3.00–3.75	4.00	3.50–4.00	P < 0.0001

**Fig. 3:**
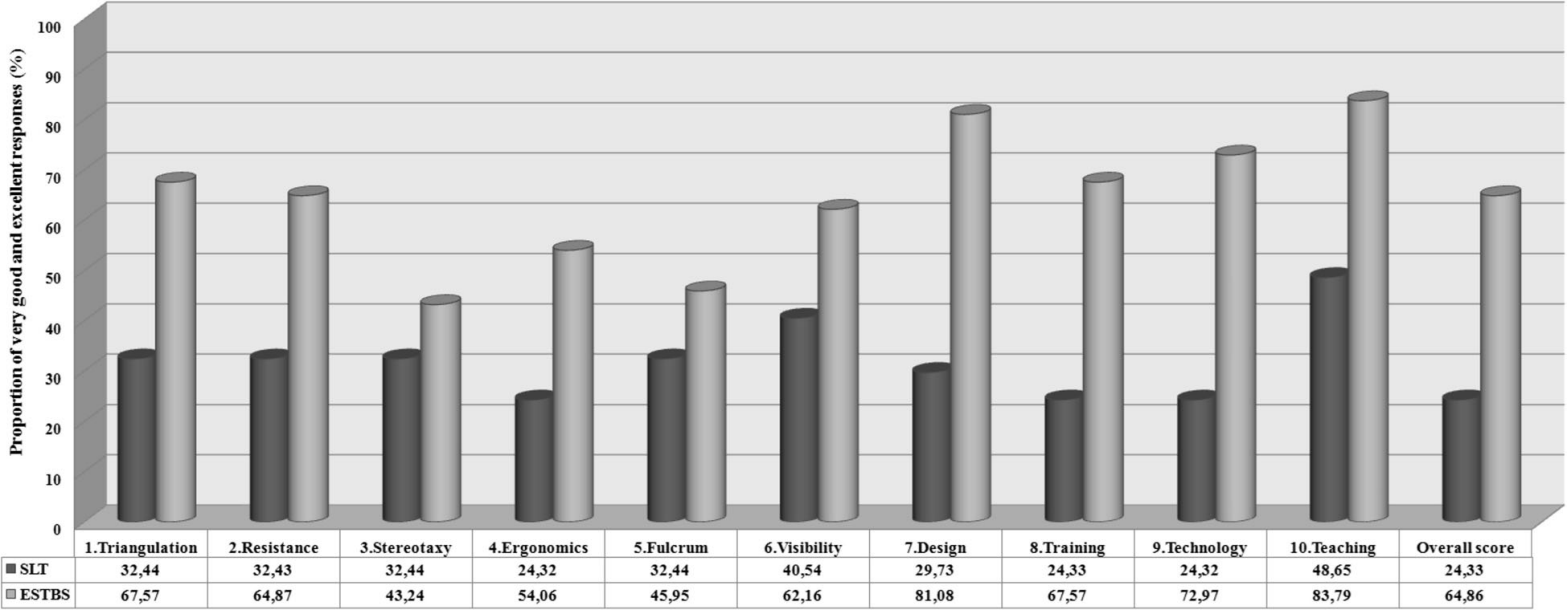
Proportion of very good (score 4) and excellent (score 5) responses given by 37 raters to EndoSuture Box Trainer Simulator (ESTBS) and Standard Laparoscopic Trainer (SLT), considering each individual statement and the overall score of the validation questionnaire

Furthermore, the proportion of very good (4) and excellent (5) scores assigned by the participants to ESTBS simulator was always greater than that attributed to SLT, considering each individual items and the overall score of the questionnaire (Fig. 3). For the items triangulation, resistance, ergonomics, design, training and technology, the proportion computed for the ESTBS was at least twice higher.

Finally, all participants were stratified according to the time spent to complete the task, providing the establishment of a Scale of Progression of Skills and Proficiency in Suture Video Surgery (Table 3), which allows classifying the trainees into 5 categories of proficiency.

**Table 3:** Scale of progression of skill and proficiency in surgical video suture

Scale	Number of surgeons (percent)
1. Insufficient (2 or 3 points)	6 (16.22)
2. Regular (4 or 5 points)	3 (8.11)
3. Good (15–18 min)	14 (37.84)
4. Very good (12–15 min)	9 (24.32)
5. Excellent (< 12 min)	5 (13.51)

## Discussion

There are several models of simulators for training in bariatric video surgery, which can be categorized into two classes: video training box and virtual simulators, according to Loukas et al. [4] The first class is a system of physical reality, where surgeons learn in inanimate models (silicone, sponges) or parts and animal organs, employing surgery video equipment, and endoscopic cameras. These simulators offer the advantage of real strength and the haptic touch since there is interaction with real instruments. The virtual simulators are purely virtual instruments which control mechanisms are integrated through appropriate sensors. The simulating software reproduces scenarios and platforms with various procedures (e.g. cholecystectomy and Nissen fundoplication operation), allowing progressive difficulty levels. However, virtual simulators are fairly criticized for high cost and low return on investment and do not reproduce important tasks like suturing.

Palter et al. [1, 5, 6] advocates that skills training using simulation promotes transfer of learning to the OR and shows to be the best method to shorten the learning curve in minimally invasive surgery, acquiring motor skills and necessary structural safety in the surgical environment. It is of significant importance to validate the teaching through systematic simulation of technical skills outside the operating room, as next step to integrate the simulation training within the curricular breadth. The educational theories can be applied within the curricular line in developing technical skills, as promote feedback, development of skills and practical proficiency training, considering various levels of difficulty, contributing, thus, to transfer of learning to the OR.

Korndorffer et al. [7] claimed to be necessary the implementation and dissemination of skill surgery laboratories (SSL). Of 253 general surgery programs in United States, 45 have the presence of this instrument. The SSL is very important to promote validation and accreditation for the training, in benefit of surgical education.

Henao et al. [8] observed an evolution effect (progression) in the surgical skills after the implementation of the laparoscopic simulator training, according to the Fundamentals of Laparoscopic Surgery (FLS) of the American Gastrointestinal Endoscopic Surgeons Society (SAGES).

Training can decrease surgical time, decreasing the risk for the patient. Torricelli et al. [9] described a laparoscopic training program for residents in urology at São Paulo School of Medicine Hospital, through critical analysis of performance and cost-benefit related to the advanced laparoscopic skill acquired in SSL. After the training, each resident of Urology was evaluated on performance of execution of 120 surgical procedures. The most common procedures performed were nephrectomy (30), suture of the bladder (30), partial nephrectomy (10), pyeloplasty (10) and others: adrenalectomy, retroperitoneoscopy, prostatectomy. These procedures are very frequent and cause low morbidity. Several training programs can be developed to minimize this potential injury and acquire proficiency in laparoscopic technique.

Kobayachi et al. [10] has developed a system of surgical training in instrumental laparoscopy, demonstrating effectiveness and construct validity, with significant improvement in performance on surgical skill validated with the evolution of a laboratory support system with low-cost design.

In the present study, the abdominal cavity ESTBS allowed various types of training and implementation of progressive complexity tasks, since the medical students, surgery residents and surgeons from several surgical areas, through continuing exercise, have advanced in their scale of progression of skills and proficiency in surgical bariatric video suture. This type of procedure is still evolving, as are the technics for obesity surgery, so the teaching of bariatric suture are continually relevant [11, 12].

The ESTBS was designed with removable top on the area equivalent to the epigastric region, to facilitate laparoscopic training steps with three-dimensional vision. With the roof closed, the vision becomes bidimentional. Two students can train simultaneously in the simulator, one as the principal surgeon and the other as an auxiliary.

The ESTBS has the potential to adapt to a video set, with two-dimensional or three-dimensional video image, which can offer high definition image and the student may have better performance in the handling of the instruments. Thus, these pictures allow making presentations that can be recorded or transmitted from a distance by teleconference. In addition, the system can be coupled to larger sized TV (50 ‘, 60 ‘or 80`), allowing theoretical-practical lessons where the teacher can do demonstrations of surgical tasks, endosutures and presentation of concepts of bariatric video surgery.

From the presentation of the supervisor, simultaneously students can follow, in others simulators, hand movements and tasks performed by the supervisor and reproduce in real time such teachings.

The study demonstrated that all parameters which evaluated the devices showed good performance for both studied simulators from a technical point of view (ergonomics, stereotaxy, visibility, strength and resilience of the grommet siliconized disk-shaped, fulcrum effect of inverted and instrument designer movements simulating a throne of the human body). However, considering teaching and training, the ESTBS demonstrated better performance.

Surgeons were stratified into specialty and area of expertise, which helped to classify the performance in execution of tasks (making the number of knots simulating a Nissen fundoplication knot tying, a medium-sized surgical procedure measured on a scale of complexity). From the data collected, it was possible to develop a Scale of Progression of Skill and Proficiency in Bariatric Suture Video Surgery, which can be used as a parameter for assessing competence in training and teaching protocols. After stratification of surgeons, it became clear the difference in time of execution of the subjected tasks, as well as the expertise, between surgical residents and young surgeons, showing that additional training time is required to advance in the development of skill and in the achievement of proficiency.

Actually, in most of Brazilian surgery residencies, training is done intraoperativelly at the OR, in real time, prolonging anestesiological and surgical time. This reality can be modified by a previous training with a simulation. In fact, endoknot, endosuturing and endoanastomosis are the most difficult maneuvers in video surgery. Safe execution of surgical video sutures broadened and may make possible the indication of laparoscopic access for performing more complex procedures.

In this study, the novel EndoSuture Trainer Box Simulator was compared to a commercially available model regarding technical, teaching and training aspects with the purpose of demonstrate its utility as a tool for bariatric surgical education. It was found that ESTBS performance was better than the reference simulator in several technical aspects, such as: the triangulation of the instruments, resistance on moving the instruments during the execution of the tasks and resiliency of the rubber silicone-grommets, the ergonomics features, the simulators design and the capacity to incorporate new technologies. Moreover, and more important, the ESTBS was also rated better concerning its ability of teaching and training surgical skills, as well as the global performance, evaluated by the overall score. These findings demonstrated the effectiveness and utility of the ESTBS as a tool for bariatric surgical education. However more physical laparoscopic simulators developments have to be pursued. This initial study appears to be promising and more randomized controlled studies are required to confirm the present results. This study had the limitations of not evaluating objective parameters. Also, the number of surgeons involved was small to draw definitive conclusions.

## Conclusions

Although laparoscopic physical simulation cannot substitute the operating room practice, it can provide a safe and cost-effective environment to enhance surgical skills, and thus allowing low and middle-income countries to implement it. The present study intended to pre-validate the novel EndoSuture Trainer Box Simulator (ESTBS) as a promising technical and technological teaching equipment. The ESTBS appears to be an effective, useful, and a low cost realistic simulator for bariatric laparoscopic training.

The performance with endosutures of residents and young surgeons at the present study pointed out to the need for continued training to acquire psycho-motor perception in this new work environment in order to absorb and expand the concepts and ascend in the Scale of Skill Progression Proficiency in Bariatric Suture Video Surgery. We expect that soon this type of experience become the commonplace of surgeon training.

## Abbreviations

ESTBS: EndoSuture Trainer Box Simulator
LED: Light-emitting diode
SLT: Standard laparoscopic trainer
SSL: Skill Surgery Laboratory
